# Supplementary material for: Humoral and T-Cell Mediated Response after the Third Dose of mRNA Vaccines in Patients with Systemic Lupus Erythematosus on Belimumab
Source: J Clin Med. 2023 Jan 30;12(3):1083. doi: 10.3390/jcm12031083 (PMC9917399; doi:10.3390/jcm12031083)
Supplement: Supplementary file 1 [file jcm-12-01083-s001.zip › jcm-2134077-supplementary.pdf]

Table S1. Demographic characteristics and immune response of the control group.

	Control 1	Control 2	Control 3	Control 4	Control 5	Control 6	Control 7	Control 8	Control 9	Control 10	Control 11	Control 12	Control 13
**Sex**	Female	Female	Female	Male	Male	Male	Female	Female	Female	Female	Male	Male	Female
**Age**	44	33	60	50	41	46	47	43	46	42	50	67	37
**Humoral and cellular response after the II dose (T0 – 2D4W)**													
Anti-RBD antibody titre, U/ml	2500	1133	1624	566	908	907	2500	1947	271	271	271	271	271
Cellular response (quantitative), pg/ml	204.8	21.85	472.46	126.09	25.87	419.03	1036.22	331	143.19	67.42	1558.51	147.71	709
Cellular response (qualitative)	Present	Present	Present	Present	Present	Present	Present	Present	Present	Present	Present	Present	Present
